# Editorial Annual Meeting of the Belgian Society of Radiology (BSR) 18th November 2017

**DOI:** 10.5334/jbr-btr.1448

**Published:** 2017-11-18

**Authors:** Didier De Surgeloose

**Affiliations:** 1 ZNA Middelheim, BE

**Figure:**
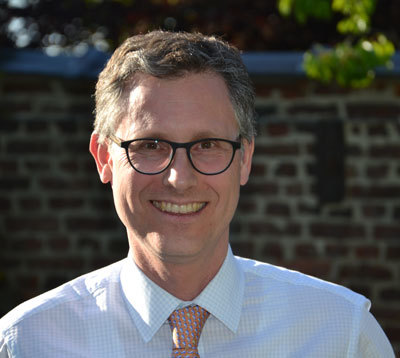
Didier De Surgeloose

This year’s annual Symposium highlights specific topics in the field of Pediatric Radiology and Neuroradiology and will again host a parallel session organized by the Young Radiologists Section. Besides dealing with useful emergency situations the lectures will be of interest to both general and specialized radiologists. The session will be chaired by Prof. Philippe Demaerel (Dutch speaking Catholic University of Leuven) and by Prof. Niloufar Sadeghi (Erasme University Hospital, ULB Brussels).

**Figure:**
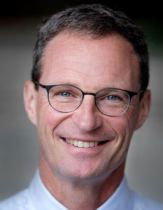
Philippe Demaerel

**Figure:**
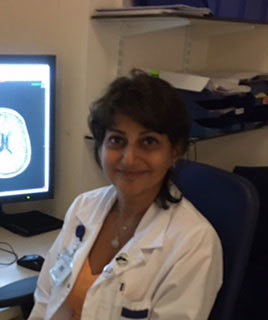
Niloufar Sadeghi

**Figure:**
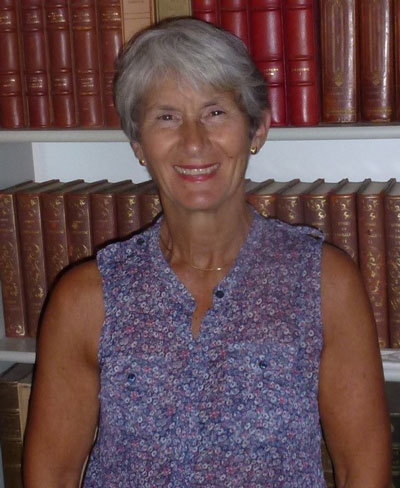
Françoise Dreyfus-Héran

The first lecture **“Imaging an Orbital Mass: the essentials”** is presented by Prof. Françoise Dreyfus-Héran, a well-known radiologist specializing in neuro-ophtalmological imaging. Dr. Héran is a full-time radiologist at the Ophtalmological Fondation Rothschild in Paris, who is a member of the SFNR Executive Committee and has beena member of the Executive Committee of the SFR IDF since 2012. She published extensively in the field of neuro-ophtalmology and salivary glands. During her lecture she will focus on adapting the technique to the clinical question and present the typical radiological aspects of orbital pathology. Attention will also be given to lesser-known techniques such as Color Doppler imaging and Diffusion-MRI.

In her lecture **“Neuroimaging of Adult Gliomas,”** Prof.Niloufar Sadeghi will explain how to deal with adult brain gliomas, a topic that is close to her heart since she dedicated her PhD thesis to biomarkers in the in-vivo evaluation of human brain gliomas in 2010. Prof. Sadeghi holds diagnostic and teaching positions at ULB-Erasme Hospital in Brussels and has recently been appointed as Head of the Clinic of Diagnostic Neuroradiology at ULB. The classification of brain tumors has evolved tremendously in recent years due to advances in research with new genetic and molecular markers. Anatomic and functional imaging now have a truly complementary role in the diagnosis of brain masses, in the planning of the surgical approach and in monitoring response to treatment.

Dr. Didier De Surgeloose, Director of the Radiology Department at ZNA Middelheim in Antwerp since 2004, will highlight the difficulties encountered when **Imaging the Spinal Cord**. Graduate of the University of Leuven (KUL) and holder of a European Diploma in Neuroradiology, Dr. Didier De Surgeloose is actively involved in adult and pediatric neuroradiology in Antwerp. Close interaction with the neurologist and knowledge of laboratory findings will guide the diagnosis in spinal cord lesions. State-of-the-art imaging of the spinal cord nowadays includes Diffusion MRI. In recent years, research led to a better understanding of complex pathologies such as MS, ADEM and Neuromyelitis Optica Spectrum Disorder (NMOSD). In NMOSD Aquaporin-4 antibodies and Myelin Oligodendrocyte Glycoprotein (MOG) antibodies present with distinct radiological and prognostic features.

**Figure:**
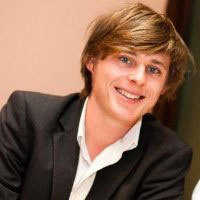
Timo De Bondt

The last lecture of this session, **“Dose optimization in CT examinations of the Brain,”** is dedicated to the necessary efforts in limiting the dose when performing CT examinations of the head. Timo De Bondt studied Physics at the University of Antwerp and wrote his PhD thesis entitled “Cyclical changes in the female brain – assessment with advanced MRI techniques” under the supervision of Prof. Paul Parizel. He coordinated the DoseWatch project at the department of Radiology of the University of Antwerp Hospital. He also completed a Master Degree in Medical Radiation Physics at the University of Leuven. In his lecture, Timo De Bondt will explain how dose management software can help radiologists to avoid excessive dosing in CT, which is of high importance especially in the pediatric population.

